# Antimycobacterial Activity of Cinnamaldehyde in a *Mycobacterium tuberculosis*(H37Ra) Model

**DOI:** 10.3390/molecules23092381

**Published:** 2018-09-18

**Authors:** Rafal Sawicki, Joanna Golus, Agata Przekora, Agnieszka Ludwiczuk, Elwira Sieniawska, Grazyna Ginalska

**Affiliations:** 1 Chair and Department of Biochemistry and Biotechnology, Medical University of Lublin, Chodzki 1, PL-20093 Lublin, Poland; joanna.golus@umlub.pl (J.G.); agata.przekora@umlub.pl (A.P.); g.ginalska@umlub.pl (G.G.); 2 Medical Plant Unit, Chair and Department of Pharmacognosy, Medical University of Lublin, Chodzki 1, PL-20093 Lublin, Poland; agnieszka.ludwiczuk@umlub.pl (A.L.); elwira.sieniawska@umlub.pl (E.S.)

**Keywords:** selectivity index, synergy, cell membrane, stress sensing, *clgR* expression

## Abstract

The purpose of the study was to evaluate the antimycobacterial activity and the possible action mode of cinnamon bark essential oil and its main constituent—cinnamaldehyde—against the *Mycobacterium tuberculosis* ATCC 25177 strain. Cinnamaldehyde was proved to be the main bioactive compound responsible for mycobacterial growth inhibition and bactericidal effects. The antimycobacterial activity of cinnamaldehyde was found to be comparable with that of ethambutol, one of the first-line anti-TB antibiotics. The selectivity index determined using cell culture studies in vitro showed a high biological potential of cinnamaldehyde. In *M. tuberculosis* cells exposed to cinnamaldehyde the cell membrane stress sensing and envelope preserving system are activated. Overexpression of *clgR* gene indicates a threat to the stability of the cell membrane and suggests a possible mechanism of action. No synergism was detected with the basic set of antibiotics used in tuberculosis treatment: ethambutol, isoniazid, streptomycin, rifampicin, and ciprofloxacin.

## 1. Introduction

The political and economic situation, as well as climate changes in many regions of the world, pushing hundreds of thousands of people to leave their homes. This changes the epidemiological situation and facilitates the spread of diseases including tuberculosis (TB). The emergence of multi-and extremely-drug-resistant *Mycobacterium tuberculosis* (Mtb) strains makes TB the global threat. Rifampicin, the last breakthrough drug, was introduced in the 1960s. The limited commercial potential for anti-TB therapies and noticeable withdrawal of pharmaceutical companies from antibacterial drug research make the situation even more disturbing.

The chemical diversity of natural sources such as essential oils (EO) is one of the areas of search for new promising mycobactericidal substances. Cinnamon essential oil has exhibited antibacterial, antifungal, antiviral, bactericidal, antiparasitics, and larvicidal activities [1,2] but little is known about its antimycobacterial characteristic. For antimycobacterial agents searching, the whole-cell screening approach has yielded significant success because it allows screening of all targets at once. The target-based compounds often do not inhibit bacterial growth because of efflux effect or poor penetration. The most challenging of whole-cell screening, however, is an identification of the target and/or action mode of the tested compound [3].

The cell membrane of *M. tuberculosis*, apart from other functions, is responsible for stress sensing and adaptive functions. The main mycobacterial envelope and stress signaling system is based on the proteins set composed of MprAB complex cooperating with the accessory sigma factor E (σ^E^). Stress dependent MprAB protein misfolding on the extracytoplasmic side of the membrane conduct stress signal by σ^E^ to the envelope-preserving ClgR-PspA-Rv2743c-Rv2742c system. The transcription factor encoded by clgR gene regulates downstream genes responsible for the cell membrane integrity protection and energy generation under stress. Except that, the ClgR controlled genes complex is necessary for MprAB-σE stress-signalling activity. This positive feedback between preserving and envelope-stress-sensing functions helps to respond to a number of cell membrane-perturbing signals during tuberculosis pathogenesis. The clgR-pspA-rv2743c-rv2742c genes set is conserved only within the *M. tuberculosis* complex consisting of *M. tuberculosis*, *M. bovis*, *M. africanum*, *M. canettii* and *M. orygis* [4,5,6].

The aim of the work was to determine the MIC and MBC values for cinnamaldehyde and cinnamon essential oil and evaluate the possible synergy of the tested substances with the panel of antibiotics currently used in the treatment of tuberculosis. Expression profile of clgR gene after exposure of *M. tuberculosis* to cinnamaldehyde shed light on its mode of action. The African green monkey kidney epithelial cells (GMK) were used for the selectivity index (SI) evaluation for both samples in cell culture studies in vitro.

## 2. Results and Discussion

The composition of the *Cinnamomumverum* bark essential oil was verified by GC/MS analysis and proved to be typical for this species. The most abundant compounds were cinnamaldehyde (75%), eugenol (7.5%), linalool (6.0%), β-caryophyllene (4.0%) and 1,8-cineole (6%). To investigate antimycobacterial properties of cinnamon EO and its main components: cinnamaldehyde, eugenol and linalool, MIC values were determined. The front-line (or primary) anti-TB drugs were used as positive control (Table 1). Determined MIC values for antibiotics were in general agreement with literature data for *M. tuberculosis* H37Ra strain [7].

Antibiotics used in MIC evaluation belong to the most important and effective medicines we have in the fight against mycobacteria. Because of this most of them have low or very low MIC values against fragile *M. tuberculosiss* trains like the H37Ra strain. Antimycobacterial activity of cinnamon EO and cinnamaldehyde was found to be one serial dilution higher than MIC for ethambutol. The same MIC for both substances and high MIC values for the next two most abundant compouds: eugenol and linalool indicate that the main constituent of cinnamon essential oil is responsible for anti-TB properties. Other authors have reported the following MIC values for cinnamaldehyde: 3.12 µg/mL (*M. tuberculosis* H37Rv), 12.5 µg/mL (*M. bovis* AN5), 24 µg/mL (*M. aviumsubsp.paratuberculosis*) [8,9]. This may suggest more universal antimycobacterial properties of cinnamaldehyde within *Mycobacterium* genus.

To differentiate between inhibiting and killing abilities of cinnamon oil and cinnamaldehyde, the MBC was determined. Both tested compounds showed the MBC equal to 32 μg/mL. To consider the compound bactericidal activity, MBC/MIC ratio must be less than or equal to 4. The calculated value was 4, what proved their strong bactericidal properties.

The same substances were examined for eventual synergism with primary anti-TB drugs. Fraction inhibitory concentration index (FIC_I_) was equal to 2 for all combinations, except cinnamon EO and streptomycin where FIC_I_ was equal to 1, therefore no synergism effect was confirmed.

To evaluate the possible cytotoxicity of cinnamaldehyde and cinnamon EO safety index (SI) was determined. Within these studies, it was demonstrated that tested natural agents only slightly reduced the viability of eukaryotic epithelial cells (GMK, also known as Vero line) after 24 h exposure. It was found that cinnamon EO and cinnamaldehyde exerted low cytotoxic effect against GMK cells and revealed significant antimycobacterial activity. Cinnamaldehyde showed significantly less cytotoxic effect than cinnamon EO and thus was characterized by higher SI value. Since promising antibacterial agents should have SI > 10, it may be inferred that cinnamaldehyde has a high biomedical potential to be used as the antimycobacterial agent (Table 2).

Other authors also identified a high biomedical potential of *C. verum* EO. Azeredoet al. demonstrated that *C. verum* EO revealed high activity against *Trypanosomacruzi* (epimastigotes, trypomastigotes, and amastigotes) and hindered the differentiation process of parasites in vitro. Similarly to our results, they also demonstrated slight cytotoxic effect of cinnamon EO against Vero cell line (green monkey kidney epithelial cells)—IC_50_ = 49.4 ± 1.12 μg/mL after 24 h exposure [2]. Whereas, Choi et al. showed relevant antibacterial activity of *C. verum* EO against *Streptococcus mutans* and *Streptococcus sobrinus* [10].

The harmful effect on cell wall and cell membrane is often used as an explanation of the antimicrobial mode of action of essential oils. For example, monoterpenes are able to function as impurities in phospholipid bilayer and introduce chaos in usually ordered structure. Since mycobacterial cell wall is lipophilic, essential oils components can easily interact with cell envelope [11]. Now Otarska et al. proved that exposure *M. aviumsubsp.paratuberculosis* to cinnamaldehyde could modify cell membrane causing the leakage of phosphate and other essential cell components and ultimately the death of cells [9].

**Figure 1:**
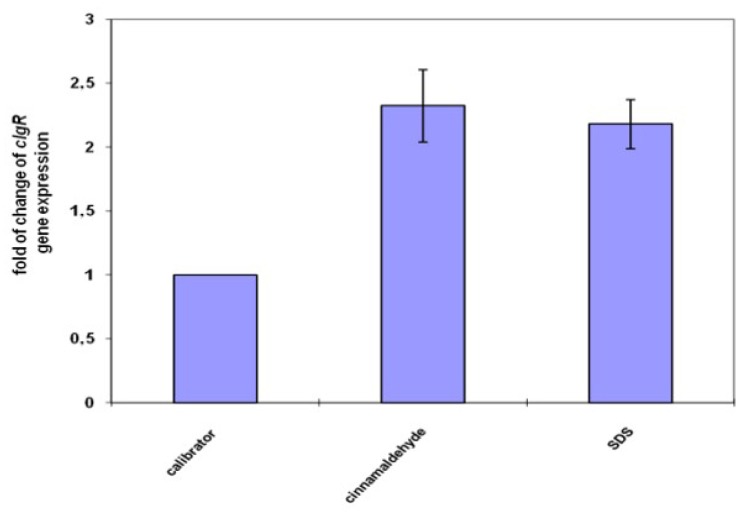
Relative expression change of *clgR* gene after 1 h exposure to the tested compound, normalized to *sigA* mRNA. Standard deviations were included as the thin bars.

The clgR gene expression was used as the marker of stress sensing/envelope preserving system status. *M. tuberculosis* exposed to the cell membrane stress agent activates two positive-feedback loops: MprAB-σE and σE-ClgR. The clgR gene encodes transcriptional factor integrating MprAB stress sensing with the envelope preserving ClgR-PspA-Rv2743c-Rv2742c system through the sigma factor σE [4]. The observed overexpression of clgR gene proves that cinnamaldehyde penetrated the thick mycobacterial cell wall and interacted with the cell membrane and/or membrane proteins (Figure 1). This finding confirmed the possible mode of action that is associated with destabilization of the mycobacterial cell membrane [12].

Concluding, cinnamaldehyde the main and active compound of *Cinnamomumverum* bark essential oil has high inhibitoryand the killing potential for *Mycobacterium tuberculosis* H37Ra. High SI index for this compound confirms its biomedical potential as the antimycobacterial agent. The *clgR* gene expression profile proves that cinnamaldehyde threatens the membrane integrity and activates stress response system. The antibiotics used in the experiment were extremely different in their mode of action but neither synergism nor antagonism was detected between tested natural agents and primary anti-TB drugs. Although cinnamaldehyde probably changes cell membrane stability/permeability it did not facilitate the action even of intracellular antibiotics like streptomycin or rifampicin. Because of its antimycobacterial properties cinnamaldehyde can be used as a starting point for the new drugs design [13,14].

## 3. Materials and Methods

### 3.1. Cinnamon Bark Oil-GC/MS Analysis

*Cinnamomumverum* bark essential oil was obtained from Unimark Remedies Ltd. (Mumbai, India). GC-MS was performed with a GC-2010 Plus gas chromatography instrument (Shimadzu Kioto, Japan) coupled to a Shimadzu QP2010 Ultra mass spectrometer. Compounds were separated on a fused silica capillary column ZB-5 MS (30 m, 0.25 mmi.d.) with a film thickness of 0.25 μm (Phenomenex, Torrance, CA, USA). Compounds were identified using a computer-supported spectral library [15,16], mass spectra of reference compounds, as well as MS data from the literature [17,18]. The identities were confirmed by comparison of retention indices with reference compounds and published data.

### 3.2. Inoculum Preparation

*M. tuberculosis* H37Ra ATCC 25177 was grown on Löwenstein-Jensen slopes (BioMaxima, Lublin, Poland) for two weeks. Bacterial mass was transferred to 7H9-S medium (Middlebrook 7H9 broth supplemented with 10% ADC-albumin dextrose catalase and 0.2% glycerol; (Becton Dickinson, Franklin Lakes, NJ, USA) and vortexed with glass beads. After 30 min of incubation for lager clumps sedimentation, upper phase was transferred to a sterile tube and left for 15 min. Next, supernatant was placed in a fresh tube and was adjusted to 0.5 McFarland standard with 7H9-S broth. The final density of inoculum in each well was approx. 5 × 10^5^ CFU/mL.

### 3.3. MIC Determination

*Cinnamomumverum* essential oil, cinnamaldehyde, eugenol, and linalool (reference standards), (Sigma-Aldrich, Saint Lois, MO, USA) were tested in the concentration range from 256 to 2 µg/mL. Serial twofold dilutions were prepared in dimethyl sulphoxide (DMSO; Sigma-Aldrich) using 7H9-Smedium as dilution. The final DMSO concentration did not exceed 1% (*v*/*v*) and had no influence on growth of the tested strain.

Isoniazid, ethambutol, rifampicin, streptomycin and ciprofloxacin (Sigma-Aldrich) were used as reference standards. Stock solutions were prepared according to the manufacturer’s instructions. Final twofold dilutions from 16 to 0.001 μg/mL were prepared in 7H9-Sbroth.

The round bottom microwell plates were prepared as follows: 50 μL of inoculum and 50 μL of tested substances were added to each well. The sterility, growth and 1% DMSO controls were included. The plates were closed with sealing foil to prevent liquid evaporation and incubated for 8 days at 37 °C. After incubation, 10 µL of resazurin (AlamarBlue, Invitrogen, Carlsbad, CA, USA) solution were added to each well, incubated for 48 h at 37 °C, and assessed for color development. The MIC was defined as the lowest drug concentration that prevented blue to pink color change [19]. The MIC determination was repeated in three independent experiments. Since the obtained results were identical, no statistical method was applied.

### 3.4. MBC Determination

Minimal bactericidal concentrations (MBCs) of *C. verum* essential oil and cinnamaldehyde against *M. tuberculosis* H37Ra were determined according to the CLSI method [20] considering longer time of *mycobacterium* incubation. After 8 days of incubation (inoculum and plates were prepared analogically to MIC assay), mycobacterium cultures were spread on agar plates (Middlebrook 7H10 agar supplemented with 10% OADC-oleic albumin dextrose catalase and 0.5% glycerol) and incubated for 4 weeks at 37 °C. MBC was defined as a compound concentration with at least a 3 log reduction of CFU compared to the initial CFU.

### 3.5. Checkerboard Assay

Synergistic interactions between *C. verum* EO/cinnamaldehyde and antibiotics: ethambutol (EMB), isoniazid (INH), streptomycin (SM), rifampicin (RMP), and ciprofloxacin (CIP) were examined by the checkerboard test based on resazurin assay similarly as MIC determination. The fractional inhibitory concentration index (FIC_I_) was calculated for each combination of two agents concentrations according to the following formula:

FIC_I_ = (MIC_A/B_/MIC_A_) + (MIC_B/A_/MIC_B_)

where MIC_A_ = MIC of the compound A alone, MIC_A/B_ is the MIC of compound A in combination with compound B and MIC_B_, MIC_B/A_ is defined analogously as compound A.

Total synergism (FIC_I_ ≤ 0.5), partial synergism (0.5 < FIC_I_ ≤ 0.75), no effect (0.75 < FIC_I_ ≤ 2) or antagonism (FIC_I_ > 2) between two compounds were deduced from the values of the FIC_I_ [21,22].

### 3.6. Cell Culture Studies In Vitro

An experiment was carried out using African green monkey kidney epithelial cells (GMK) obtained from BIOMED-Lublin S.A. (Lublin, Poland). To assess the cytotoxicity of the tested agents, the GMK cells were seeded in 96-multiwell plates at a concentration of 3 × 10^4^ cells/well (in 100 μL of the complete culture medium) and the plates were maintained at 37 °C for 24 h. Then, the culture medium was discarded and replaced with 100 µL of culture medium containing different concentrations of cinnamon EO, cinnamaldehyde, and a reference antimycobacterial drug-rifampicin. The compounds were tested at 9 different concentrations (in the range 500–1.95 µg/mL). After 24 h exposure to the tested compounds and reference drug, viability of GMK cells was assessed using MTT assay (Sigma-Aldrich) as described earlier [23]. The test was carried out in 3 independent experiments (*n* = 3) and performed in octuplicate. The IC_50_ values (concentration reducing cell viability by 50%) were determined using GraphPad Prism 5 (GraphPad Software, La Jolla, CA, USA). Based on obtained MIC values against *M. tuberculosis* H37Ra and IC_50_ determined using GMK cells, the selectivity index (SI) in vitro was calculated using the following formula:

SI = IC_50_/MIC.

### 3.7. Exposure to Cinnamaldehyde

Freshly prepared 50 mL of 7H9-S were inoculated with 1 mL of *M. tuberculosis* H37Ra of 1 McFarland standard. Bacteria were incubated at 37 °C with gentle aeration (100 RPM) for three weeks. Then, 8 mL of culture were moved to a fresh tube and exposed for 1 h to 512 µg/mL cinnamaldehyde (value determined by resazurin assay for the short 1-h exposure for late log-phase of mycobacterial culture). Culture without tested compound supplementation (8 mL) was used as expression level control. As the positive control, additional 8 mL of culture supplemented with SDS (final concentration 0.03% (300 µg/mL)) were added and samples were incubated for 1 h accordingly to the elegant experiment described by Datta at all. [4]. After incubation, tubes were centrifuged and total mycobacterial RNA was isolated.

#### 3.7.1. Total RNA Preparation

Total RNA isolation was performed with Fast RNA Pro Blue Kit (MP Biomedicals, Santa Ana, CA, USA) according to manufacturer’s instructions. Cells were disrupted in a bead-beater FastPrep24 ((MP Biomedicals, Santa Ana, CA, USA). Possible RNA contamination with genomic DNA was eliminated with deoxyribonuclease I (Sigma-Aldrich) according to manufacturer’s manual. The RNA concentration and purity were measured spectrophotometrically, aliquoted and stored at −80 °C for further use.

#### 3.7.2. qPCRReaction

All quantitative PCR (qPCR) reactions ware done with LightCycler^®^ EvoScript RNA SYBR^®^ Green I Master kit (Roche, Penzberg, Germany) in LightCycler^®^ 480 thermal cycler (Roche) in total volume of 20 μL per reaction on 96 (Roche, Penzberg, Germany). Reactions were managed with automatic pipetting station epMotion 5070 (Eppendorf, Hamburg, Germany).

The PCR efficiency was measured for each pair of primers used inexperiments. For relative quantification of transcript levels, target gene was normalized to the housekeeping gene *sigA.* The *clgR* primers (fwd5′AGCTGCAGAGCCGTACAAAT3′, rev 5′CCTCGGGTATCTGTCGGAGA3′, calculated efficiency 2.02) and *sigA* (fwd5′GACGAAGACCACGAAGAC3′, rev 5′TCATCCCAGACGAAATCAC3′, calculated efficiency 1.98) were synthesized by Sigma-Aldrich. Relative quantification was calculated according to Pfaffl mathematical model [24]. The test was carried out in 2 independent experiments and performed in triplicate.

## Figures and Tables

**Table 1:** The MIC values of antibiotics, *Cinnamomumverum* EO, cinnamaldehyde, eugnol and linalool determined for *M. tuberculosis* H37Ra.

Tested Agent	MIC (μg/mL)
Rifampicin	0.002
Ciprofloxacin	0.125
Isoniazid	0.25
Streptomycin	0.5
Ethambutol	4
*Cinnamomumverum* EO	8
Cinnamaldehyde	8
Eugenol	256
Linalool	>256

**Table 2:** IC_50_ and SI values determined for *Cinnamomumverum* EO, cinnamaldehyde, and rifampicin as reference drug.

Tested Agent	MIC (μg/mL)	IC_50_ (μg/mL)	SI
*Cinnamonumverum* EO	8	69.35 ± 1.05	8.67
cinnamaldehyde	8	112.6 ± 1.04	14.08
rifampicin	0.002	170.2 ± 2.5	85.10
